# Labeling the oily core of nanocapsules and lipid-core nanocapsules with a triglyceride conjugated to a fluorescent dye as a strategy to particle tracking in biological studies

**DOI:** 10.1186/1556-276X-9-233

**Published:** 2014-05-13

**Authors:** Luana Almeida Fiel, Renata Vidor Contri, Juliane Freitas Bica, Fabrício Figueiró, Ana Maria Oliveira Battastini, Sílvia Stanisçuaski Guterres, Adriana Raffin Pohlmann

**Affiliations:** 1 Pós-Graduação em Ciências Farmacêuticas, Faculdade de Farmácia, Universidade Federal do Rio Grande do Sul, Av. Ipiranga, 2752, Porto Alegre, RS 90610-000, Brazil; 2 Departamento de Química Orgânica, Instituto de Química, Universidade Federal do Rio Grande do Sul, Av. Bento Gonçalves, 9500, Porto Alegre, RS 91501-970, Brazil; 3 Departamento de Bioquímica, Instituto de Ciências Básicas da Saúde, Universidade Federal do Rio Grande do Sul, Rua Ramiro Barcelos, 2600, Porto Alegre, RS 90035-003, Brazil

**Keywords:** Fluorescent triglyceride, Fluorescent polymeric nanocapsules, Lipid-core nanocapsules, Fluorescence, Cell uptake

## Abstract

The synthesis of novel fluorescent materials represents a very important step to obtain labeled nanoformulations in order to evaluate their biological behavior. The strategy of conjugating a fluorescent dye with triacylglycerol allows that either particles differing regarding supramolecular structure, i.e., nanoemulsions, nanocapsules, lipid-core nanocapsules, or surface charge, i.e., cationic nanocapsules and anionic nanocapsules, can be tracked using the same labeled material. In this way, a rhodamine B-conjugated triglyceride was obtained to prepare fluorescent polymeric nanocapsules. Different formulations were obtained, nanocapsules (NC) or lipid-core nanocapsules (LNC), using the labeled oil and Eudragit RS100, Eudragit S100, or poly(caprolactone) (PCL), respectively. The rhodamine B was coupled with the ricinolein by activating the carboxylic function using a carbodiimide derivative. Thin layer chromatography, proton nuclear magnetic resonance (^1^H-NMR), Fourier transform infrared spectroscopy (FTIR), UV-vis, and fluorescence spectroscopy were used to identify the new product. Fluorescent nanocapsule aqueous suspensions were prepared by the solvent displacement method. Their pH values were 4.6 (NC-RS100), 3.5 (NC-S100), and 5.0 (LNC-PCL). The volume-weighted mean diameter (*D*_4.3_) and polydispersity values were 150 nm and 1.05 (NC-RS100), 350 nm and 2.28 (NC-S100), and 270 nm and 1.67 (LNC-PCL). The mean diameters determined by photon correlation spectroscopy (PCS) (*z*-average) were around 200 nm. The zeta potential values were +5.85 mV (NC-RS100), -21.12 mV (NC-S100), and -19.25 mV (LNC-PCL). The wavelengths of maximum fluorescence emission were 567 nm (NC-RS100 and LNC-PCL) and 574 nm (NC-S100). Fluorescence microscopy was used to evaluate the cell uptake (human macrophage cell line) of the fluorescent nanocapsules in order to show the applicability of the approach. When the cells were treated with the fluorescent nanocapsules, red emission was detected around the cell nucleus. We demonstrated that the rhodamine B-conjugated triglyceride is a promising new material to obtain versatile dye-labeled nanocarriers presenting different chemical nature in their surfaces.

## Background

Polymeric nanocapsules, which are nanoscale particles prepared by self-assembling methods and composed of a polymeric wall surrounding an oily core, have been studied to direct drugs toward their targeted therapeutic site of action [1-4]. Due to the lipophilic core, the entrapment of hydrophobic drugs in nanocapsules is more efficient in comparison with polymeric nanospheres [1,5]. In addition, nanocapsules are more suitable for prolonged release during the sustained phase [6]. Polymeric nanocapsules are referred to as lipid-core nanocapsules when sorbitan monostearate is used together with the triacylglycerol to prepare the nanocapsules forming an organogel as core [7-9]. In general, when an active substance is entrapped in a carrier, the mechanism of action is not only dependent on the interactions of the substance with the cells and/or tissues but also on the behavior of the carrier within the organism [10].

The fluorescence phenomenon involves the absorption of light at a particular wavelength and the emission of electromagnetic radiation at higher wavelengths, in the near ultraviolet-visible region, which makes it a technique of high sensitivity where very low concentrations can be detected [10]. Fluorescent techniques can be applied to verify the location of the nanoparticles within cells or their mechanisms of interaction with cells or tissues [11-15]. For this purpose, a fluorescent dye must be physically entrapped within [16,17] or chemically bound to [12,18,19] the nanocarriers. In the latter case, greater stability of the dye-particle complex can be achieved, and the kinetics of the dye release from the particle should be slower, reducing the possibility of false results.

Therefore, the synthesis of the fluorescent materials used to prepare nanoformulations represents a very important step in relation to evaluating their biological behavior. With regard to the use of fluorescent dyes chemically bound to nanocapsules, few studies involving the labeling of the polymeric wall are available [12,18,20-22]. In this regard, it should be noted that, depending on the chemical characteristics of the polymer, labeling the polymer used to prepare the particles with a fluorescent dye can change the surface nature of the nanocarrier. The alternative of labeling a triacylglycerol can allow the obtainment of diverse fluorescent dye-labeled nanocarriers such as nanoemulsions, nanostructured lipid carriers, polymeric nanocapsules, and lipid-core nanocapsules. Additionally, by labeling the lipophilic core, versatile nanocarriers can be obtained, non-ionic, cationic, or anionic polymeric nanocapsules. Rhodamine B was chosen as the fluorescent dye for use in this study, due to the high fluorescence quantum efficiency and low cost. Castor oil (CAO) was chosen as the reactant since its major component, ricinolein, has three hydroxyl groups in its molecule which can react with the carboxyl group of rhodamine B. In order to study whether fluorescent nanoparticles with different surface characteristics could be obtained, the novel fluorescent product was the core material of Eudragit RS100 or Eudragit S100 nanocapsules (NC), which have cationic and anionic surfaces, respectively. To verify if different supramolecular structure could also be obtained, fluorescent lipid-core nanocapsules (LNC) were prepared using sorbitan monostearate and the novel rhodamine B triacylglycerol conjugate as core and poly(ϵ-caprolactone) as interfacial polymer. To investigate if the fluorescent-labeled NC and LNC could be observed by fluorescence microscopy, the nanoparticle uptake was evaluated using a human macrophage cell line.

## Methods

### Materials

Castor oil was kindly donated by Campestre (São Bernardo do Campo, Brazil). Eudragit S100® and Eudragit RS100® were obtained from Almapal (São Paulo, Brazil). Rhodamine B, 4-(*N*,*N*-dimethyl)aminopyridine (DMAP), 1-ethyl-3-(3-dimethylaminopropyl)carbodiimide hydrochloride (EDCI.HCl), poly(ϵ-caprolactone) with weight average molar mass (Mw) of 14 kg mol^-1^ (PCL_14_), sorbitan monostearate (Span® 60), and phorbol 12-myristate 13-acetate (PMA) were purchased from Sigma-Aldrich (Sao Paulo, Brazil). Poly(ϵ-caprolactone) with Mw = 116 kg mol^-1^ (Capa^TM^ 6500) (PCL_116_) was kindly donated by Perstorp (Toledo, OH, USA). Capric/caprylic triglyceride (CCT) was acquired from Alpha Quimica (Porto Alegre, Brazil). Polysorbate 80 and sorbitan monooleate (Span 80®) were supplied by Delaware (Porto Alegre, Brazil). RPMI 1640, penicillin/streptomycin, Fungizone®, and 0.5% trypsin/EDTA solution were obtained from Gibco (Gibco BRL, Carlsbad, CA, USA). Fetal bovine serum (FBS) was obtained from Cultilab (Cultilab, Campinas, SP, Brazil). UltraCruz® mounting medium for fluorescence studies with DAPI was supplied by Santa Cruz Biotechnology, Inc. (Santa Cruz, CA, USA). The acetonitrile (ACN) used in the fluorescence measurements was spectroscopic grade. All other reagents were of analytical grade. Reagents and solvents were used as received, with the exception of dichloromethane, which was distilled after drying with calcium hydride under reflux.

### Synthesis and characterization of rhodamine B-labeled triglyceride (1)

CAO, whose main component is ricinolein (the triglyceride of ricinoleic acid, approximately 90%) [23], was covalently coupled with a fluorescent dye, rhodamine B (RhoB). Briefly, rhodamine B (1.91 g) and DMAP (0.49 g) were dissolved in dry dichloromethane (30 mL) at room temperature under argon. After 40 min of stirring, EDCI.HCl (0.82 g) dissolved in dry dichloromethane (12 mL) was added to the reaction medium cooled in an ice bath. After 40 min under stirring, the CAO (2.08 g) dissolved in dry dichloromethane (4 mL) was then added. The reaction medium was kept under stirring for 2 days in an argon atmosphere at room temperature. After this period, dichloromethane (30 mL) was added to the organic phase, and the extraction was carried out with aqueous solutions of firstly 1 mol L^-1^ HCl (3 × 40 mL) and then saturated NaHCO_3_ (3 × 40 mL). The organic phase was extracted with water (6 × 40 mL), dried under magnesium sulfate anhydrous, filtered, and evaporated under reduced pressure. The fluorescent product was purified by column chromatography using silica gel (60 to 200 mesh) and CHCl_3_ as eluent. The product 1 was obtained as an oil. After purification, the process yielded 1.0 g of product 1.

The product 1 was characterized by thin layer chromatography (TLC), Fourier transform infrared spectroscopy (FTIR), proton nuclear magnetic resonance (^1^H-NMR), size exclusion chromatography (SEC), UV-vis spectroscopy, and spectrofluorimetry. The TLC was performed using dichloromethane/methanol (9:1, *v*/*v*) as eluent and an aluminum sheet (Merck, Whitehouse Station, NJ, USA) covered with silica gel 60 (70 to 230 mesh) as stationary phase. The bands were revealed under UV light at 365 nm (BOIT-LUB01, Boitton, Brazil). FTIR spectra were recorded on a Varian® 640-IR spectrophotometer (Palo Alto, CA, USA) from 4,000 to 400 cm^-1^ (100 scans, 2 cm^-1^ resolution), using sodium chloride crystals. FTIR: 3,390 cm^-1^ (OH stretching), 2,940 and 2,850 cm^-1^ (CH_2_, asymmetric and symmetric stretching), and 1,740 cm^-1^ [C = O (ester)].

SEC analysis was carried out using a Viscotek® VE 2001 chromatograph with a Viscotek® TDA 302 triple detector and PS/DVB column (Malvern Instruments, Westborough, MA, USA). The purified product 1 and raw castor oil were dissolved in tetrahydrofurane, filtered (0.45 μm), and analyzed using polystyrene as reference. The product 1 was diluted in ACN and the maximum absorption wavelength (*λ*_ab_) was evaluated by UV-vis spectroscopy using a spectrophotometer (Shimadzu® UV-1601PC, Nakagyo-ku, Kyoto, Japan). The *λ*_ab_ value was used to determine the maximum emission wavelength (*λ*_max*-*em_) by fluorimetry with a spectrofluorometer (Cary® 100, Agilent, Santa Clara, CA, USA).

The amount of rhodamine B residues chemically bound to ricinolein presented in the product 1 was quantified by the standard addition method using a UV-visible spectrofluorometer (Cary® 100, Agilent). Firstly, an ethanol solution of RhoB was prepared (2.25 μmol L^-1^) and aliquots of this solution were diluted with ACN in volumetric flasks. Calibration curves were constructed within a range of 0.108 to 0.539 μmol L^-1^. A fixed concentration of the product 1 (0.152 mg mL^-1^) was maintained in all samples used to construct the calibration curve. The fluorescence intensity (*I*_f_) was measured using a rectangular cuvette (Hellma Quartz Suprasil®, 10 mm, Sigma-Aldrich) with the maximum excitation (*λ*_max-ex_) and *λ*_em_ wavelengths observed for the product 1. The *I*_f_ was plotted as a function of the molar concentration of rhodamine B. The linear coefficient value for the linear regression corresponded to the amount of RhoB presented in purified product 1. The experiment was replicated three times.

### Preparation of the fluorescent nanocapsules

The fluorescent-labeled polymeric nanocapsules were prepared by the solvent displacement method [8,24]. The polymers Eudragit RS100 and Eudragit S100 were used to prepare the nanocapsule formulations NC-RS100 [25] and NC-S100 [26], respectively, and the polymer poly(ϵ-caprolactone) (PCL) was used to obtain the lipid-core nanocapsule formulation LNC-PCL [27]. To prepare the nanocapsule formulations (NC-RS100 and NC-S100), an organic phase (27 mL of acetone), containing the polymer (100.0 mg), CCT/product 1 (9:1, *w*/*w*) (333 μL), and sorbitan monooleate (76.6 mg) (except for NC-RS100), was injected using moderate stirring into a polysorbate 80 aqueous phase (76.6 mg in 53 mL). The organic solvent was removed by evaporating the suspension under reduced pressure. The suspension was evaporated until a final volume of 10 mL. The LNC-PCL formulation was obtained by the same procedure. However, in this case, the organic phase was composed of the polymers, PCL116 (90.0 mg) and PCL14 (10.0 mg), CCT/product 1 (9:1, *w*/*w*) (160 μL), and sorbitan monostearate (40.0 mg) dissolved in acetone (27 mL). Three batches of each formulation were prepared.

### Characterization of the fluorescent-labeled nanocapsules

The pH of the formulations was measured without dilution of the suspensions using a potentiometer, model B474 (Micronal, Brazil). Laser diffraction analysis was performed with a Malvern Mastersizer® 2000 instrument (Malvern Instruments, Worcestershire, UK) and used to determine the particle size distribution profile, volume-weighted mean diameter (*D*_4.3_), and polydispersity (SPAN). Photon correlation spectroscopy (PCS) was used to characterize the nanometric population by determining the average diameter (*z*-average) and polydispersity index. Electrophoretic mobility (EM) analysis was performed to determine the zeta potential values. PCS and EM analyses were performed using a ZetaSizer ZS (Malvern), and the formulations were previously diluted (500-fold) in pre-filtered ultrapure water or a 10 mM NaCl solution (pH = 5.65 ± 0.07), respectively.

The concentration of particles (particles per mL) in each formulation was evaluated by nanoparticle tracking analysis (NTA) with a NanoSight LM10 system (NanoSight, Amesbury, UK), equipped with a sample chamber and a 640-nm laser. For the analysis, the formulations were diluted (5,000-fold) in ultrapure water to obtain samples with 10^8^ to 10^9^ particles per mL and injected into the sample chamber with a syringe. Having in mind that NTA analysis can lack in quality of results when polydisperse systems are analyzed, the same parameters were used for the records and process of each sample. The records were taken over 60 s using a camera shutter of 207 and gain of 177. The data were subsequently analyzed using NTA 2.3 Build 0011 RC1 software (gain of 1.56, blur of 3 × 3, and min particle size of 50 nm). Particles moving under Brownian motion are identified and tracked individually by the software which gives the particle concentration of the sample.

The fluorescence spectra of the formulations were investigated by fluorimetry with direct analysis or after diluting (10-fold) in ACN (1 mL of the formulation in 10 mL of acetonitrile) using triangular rectangular cuvettes (Hellma Quartz Suprasil®, 10 mm, Sigma-Aldrich) for the measurements. For comparison purposes, samples containing 160 μL (same quantity contained in 10 mL of the LNC-PCL formulation) or 333 μL (same quantity contained in 10 mL of the NC-RS100 or NC-S100 formulation) of the mixture of CCT/product 1 (9:1, *w*/*w*) in 10 mL of ACN were analyzed to obtain their fluorescence profiles. These samples were then diluted (10-fold) and analyzed.

### Fluorescence microscopy

A human macrophage cell line was used as the cell model to evaluate the fluorescent nanoparticle uptake. The human monocytic U937 cell line was cultured in suspension in RPMI medium supplemented with 10% FBS at 37°C under a 5% CO_2_ atmosphere. The cells were differentiated into macrophages by seeding the cells, at a density of 5 × 10^4^ cells per circular cover slip (diameter = 13 mm) (Glasscyto, Brazil), and placing them into each plate well (24-well plate), with resuspension in U937 medium and supplementation with 10 nM PMA for 3 days at 37°C under 5% CO_2_ atmosphere. After this period, the medium was removed and the adherent cells were treated with the fluorescent nanoparticles (5 μL for NC-RS100 and NC-S100 formulations and 10 μL for LNC-PCL formulation), diluted in RPMI medium (500 μL), corresponding to a density of approximately 4.3 to 6.5 × 10^10^ particles per mL (approximately 3.15 μg mL^-1^ of product 1) per well containing the cover slip, and incubated for 2 h. A control group did not receive any treatment. The cells were then washed twice with PBS, fixed with a 2% glutaraldehyde/4% paraformaldehyde solution (20 min), and again washed twice with PBS. The cover slips were placed (cells down) on microscope slides containing 10 μL of mounting medium with DAPI to stain the cell nucleus. Cellular imaging was carried out with a Nikon eclipse TE300 inverted fluorescent microscope (Nikon, Tokyo, Japan) (×200 magnification) equipped with a digital camera. Standard filters for DAPI (blue) or rhodamine (red) were used. The images were processed using the ImageJ program, applying the same setting parameters (brightness and contrast) to all samples, aiming to improve the blue and red fluorescence intensity. The overlap of the channels (red and blue) was achieved using the BioImageXD program.

## Results

### Synthesis of the product 1

**Figure 1:**
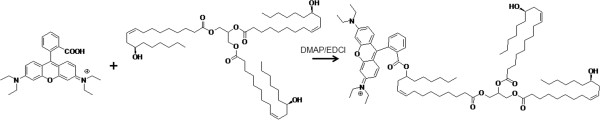
**General reaction scheme.** Rhodamine B coupling with hydroxyl group of ricinolein contained in the castor oil using DMAP and EDCI in dichloromethane to produce product 1.

**Figure 2:**
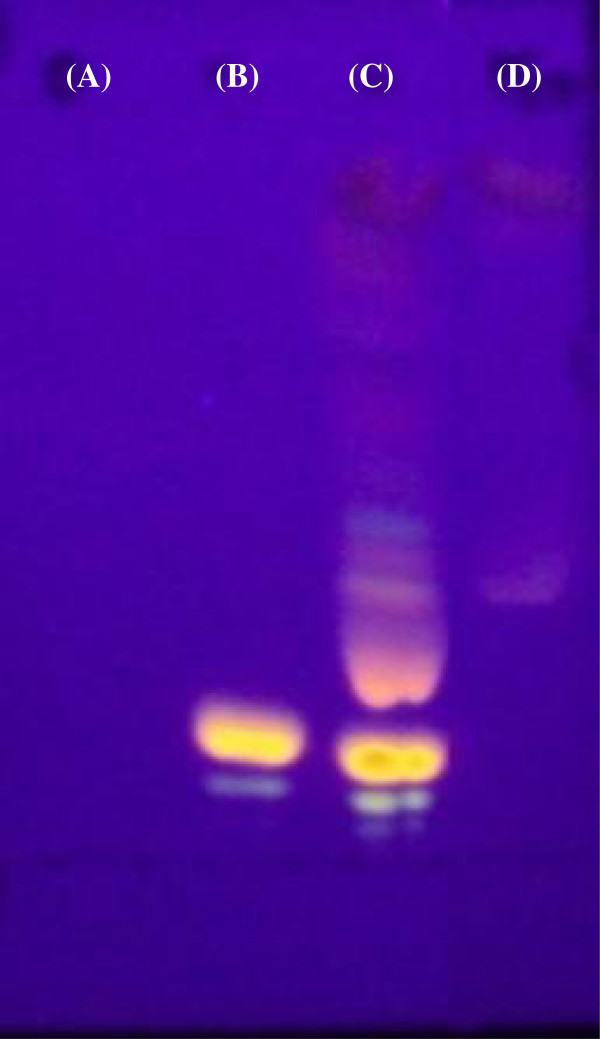
**Thin layer chromatography (TLC) image. (A)** Raw castor oil, **(B)** rhodamine B, **(C)** crude fluorescent product 1, and **(D)** purified fluorescent product 1.

The product 1 was obtained as a brilliant orange oily product after the reaction of the vegetable oil with rhodamine B in the presence of EDCI and DMAP (Figure 1) followed by purification through column chromatography. The TLC image in Figure 2 shows spots of CAO (a), rhodamine B (b), the crude fluorescent product 1 (c), and the purified fraction of the fluorescent product 1 (d) after revelation with UV light. As expected, the CAO spot was not revealed. Rhodamine B eluted with a retention factor (*R*_f_) of 0.14. Besides the characteristic spot of RhoB, several other spots can be observed for the elution of the crude product 1 (c). No spot presenting the *R*_f_ of RhoB was observed for the purified product 1 (d).

**Figure 3:**
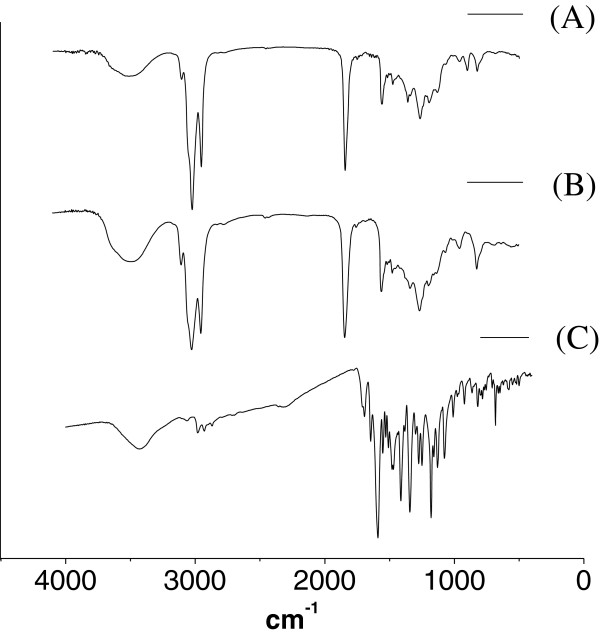
**Infrared spectra. (A)** purified product 1 (product 1), **(B)** raw castor oil (CAO), and **(C)** rhodamine B (RhoB).

FTIR spectra of the starting raw materials of the reaction (CAO and RhoB), as well as of the purified fluorescent product 1, are shown in Figure 3. The product 1 (Figure 3 (A)) and CAO (Figure 3 (B)) showed similar FTIR spectra. However, in the FTIR spectrum for the product 1 (Figure 3 (A)), no band was observed at 1,595 cm^-1^ [C = O (carboxylic acid)] in contrast to the spectrum for the raw RhoB, in which this peak was present (Figure 3 (C)). Regarding the ^1^H-NMR spectrum, signals with a chemical shift at low field (*δ* = 5.9 to 7) were observed only for the fluorescent product 1.

**Figure 4:**
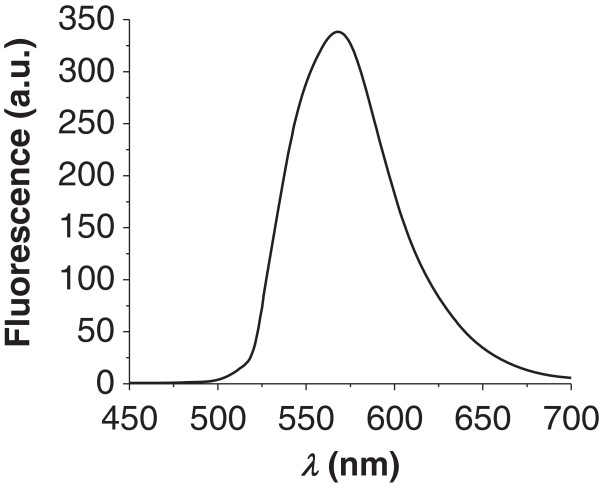
**Fluorescence emission spectrum of the synthesized product 1 (1.52 mg mL**^**-1**^**).**

The UV-vis spectrum for the purified product 1 showed *λ*_max*-*ab_ at 519 nm. The spectrofluorimetry analysis was then performed using the above-mentioned wavelength for excitation of the samples. The emission spectrum for a sample containing 1.52 mg mL^-1^ of the fluorescent product 1 presented *λ*_max*-*em_ at 567 nm with an intensity of 340 a.u. (Figure 4). Quantification of rhodamine B bound to the rhodamine-labeled triglyceride (product 1) was performed using the standard addition method (*r* > 0.99) indicating a concentration of bound dye of 0.517 ± 0.096 μmol per g of product 1.

The values for the Mw and number average molar mass (Mn) were found to be 2,108 and 1,645 g mol^-1^, respectively, obtained through SEC analysis of the purified product 1. For the raw castor oil sample, the corresponding values were 2,120 g mol^-1^ (Mw) and 1,834 g mol^-1^ (Mn).

### Characterization of the fluorescent nanocapsules and fluorescent lipid-core nanocapsules

**Figure 5:**
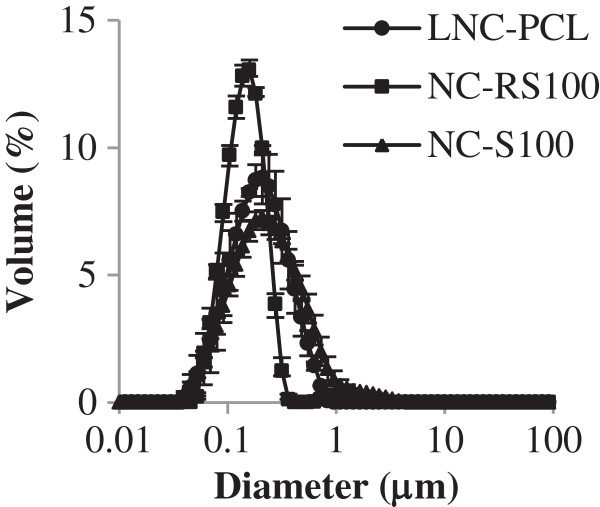
**Particle size distribution profiles by volume obtained using laser diffraction (mean ± SD, *n*** **= 3).**

After their preparation, the pH values obtained for the formulations were around 4.6 (NC-RS100), 3.5 (NC-S100), and 5.0 (LNC-PCL) (Table 1). Laser diffraction analysis indicated a size distribution profile with the major particle size fraction in the nanometer scale for all formulations (Figure 5). The NC-S100 formulation presented a small fraction of particles in the micrometer scale by volume (Figure 5).

**Table 1:** **Physicochemical characterization of the formulations (mean ± SD, *n*** **= 3)**

**Sample**	**pH**	***D***_**4.3 **_**(nm)**	**SPAN**	***z*-average (nm)**	**PDI**	**ZP (mV)**
LNC-PCL	4.91 ± 0.12	270 ± 85	1.67 ± 0.10	198 ± 8	0.10 ± 0.02	-19.25 ± 4.16
NC-RS100	4.60 ± 0.11	146 ± 9	1.05 ± 0.07	170 ± 25	0.15 ± 0.08	+5.85 ± 0.56
NC-S100	3.50 ± 0.09	344 ± 14	2.28 ± 0.03	207 ± 28	0.21 ± 0.13	-21.12 ± 6.45

The *D*_4.3_ values observed for the nanoformulations were around 150 nm (NC-RS100), 350 nm (NC-S100), and 270 nm (LNC-PCL) (Table 1). SPAN values of 1.05 (NC-RS100), 2.28 (NC-S100), and 1.67 (LNC-PCL) were obtained. The mean diameters of the formulations measured by PCS (*z*-average) were close to 200 nm with polydispersity index (PDI) values lower than 0.34. The zeta potential values were negative for the NC-S100 and LNC-PCL formulations and positive for the NC-RS100 formulation. The concentrations of particles per mL for each formulation were 5.56 ± 0.15 × 10^12^ particles (NC-RS100), 4.35 ± 0.41 × 10^12^ particles (NC-S100), and 3.22 ± 0.58 × 10^12^ particles (LNC-PCL).

**Figure 6:**
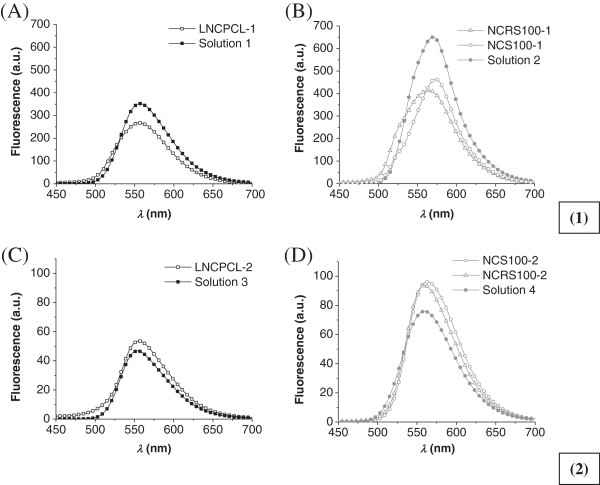
**Fluorescence emission spectra of samples.** (1) Fluorescence emission spectra of undiluted/unextracted samples of the formulations: **(A)** LNC-PCL-1 compared to solution containing 160 μL (solution 1) of the CCT/fluorescent triglyceride product 1 mixture in 10 mL of ACN and **(B)** NC-RS100-1 and NC-S100-1 compared to solution containing 333 μL (solution 2) of the CCT/fluorescent triglyceride product 1 mixture in 10 mL of ACN. (2) Fluorescence emission spectra of diluted/extracted samples (10-fold in ACN) of the formulations: **(C)** LNC-PCL-2 compared to diluted solution (10-fold) of solution 1 (solution 3) and **(D)** NC-RS100-2 and NC-S100-2 compared to diluted solution (10-fold) of solution 2 (solution 4).

Figure 6 shows the fluorescence emission spectra obtained for samples of the undiluted/unextracted (Figure 6A,B) and diluted/extracted (Figure 6C,D) formulations. Solutions containing the same quantities of the CCT/fluorescent product 1 mixture as those in the LNC-PCL (solution 1) or NC-RS100 and NC-S100 (solution 2) formulations presented an *λ*_max-em_ value of 567 nm, with fluorescence intensities of 346 and 642 a.u., respectively (Figure 6A,B). Concentrated samples of the formulations NC-RS100 and LNC-PCL (NC-RS100-1 and LNC-PCL-2) presented an *λ*_max-em_ value of 567 nm with intensities of 412 and 232 a.u., respectively, while for NC-S100 (NC-S100-1), this value was shifted to a higher wavelength (574 nm) compared to that of the CCT/fluorescent product 1 mixture (9:1, *w*/*w*) with an intensity of 464 nm (Figure 6A,B).

The *λ*_max-em_/*I*_*f*_ values for the diluted solutions (solution 3 and solution 4) of the primary solutions 1 and 2, respectively, of the CCT/fluorescent product 1 mixture were 567 nm/40 a.u. (solution 3) and 567 nm/75 a.u. (solution 4) (Figure 6C,D). After diluting the nanocapsules and lipid-core nanocapsule suspensions with ACN to extract the fluorescent product 1, the NC-RS100 and LNC-PCL samples (NC-RS100-2 and LNC-PCL-2) maintained the value of *λ*_max-em_ = 567 nm with fluorescence intensities of 99 and 45 a.u., respectively. The diluted/extracted NC-S100 sample (NC-S100-2) presented *λ*_max-em_/*I*_*f*_ values of 569 nm/102 a.u.

### Fluorescence microscopy

**Figure 7:**
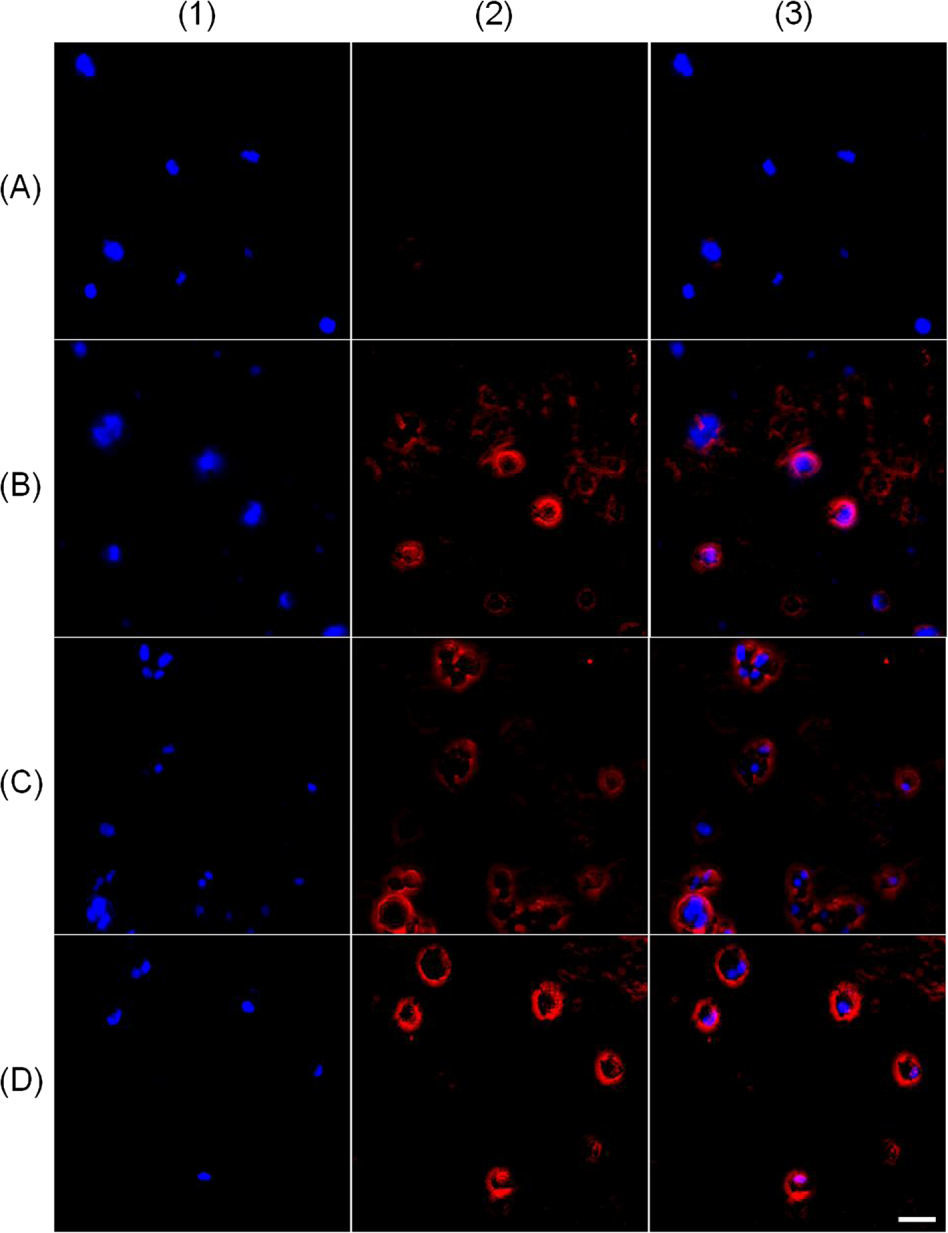
**Fluorescence microscopy images (magnification × 200) after the cell uptake study.** Macrophage cells **(A)** with no treatment and after treatment with **(B)** NC-RS100, **(C)** LNC**-**PCL, and **(D)** NC**-**S100. (1) Blue channel, (2) red channel, and (3) blue**-**red channel overlay. White scale bar in *D*_3_ = 80 μm.

A cell uptake study was carried out to investigate the potential for the fluorescence of the fluorescent nanoparticles to be used for localization in biological studies. As demonstrated in the fluorescence characterization of the fluorescent triglyceride-labeled nanocapsules and fluorescent triglyceride-labeled lipid-core nanocapsules, the particles containing the fluorescent triglyceride (product 1) presented red fluorescence (rhodamine B). The cell nucleus appears in blue (DAPI). After 2 h of incubation, red fluorescence was detected in the cells treated with the fluorescent particles (NC-RS100, LNC-PCL, and NC-S100) (Figure 7B,C,D). Fluorescence was not detected in the cells that did not receive fluorescent nanocapsules (control group) (Figure 7A).

## Discussion

A rhodamine B-labeled triglyceride (product 1) was obtained in order to prepare fluorescent nanocapsules with different properties, such as anionic or cationic surfaces, achieved by changing the polymer used to prepare the nanocarrier. Fluorescent LNC were also prepared.The RhoB carboxyl group was activated by a carbodiimide. This intermediate product reacted with the hydroxyl groups of ricinolein, contained in the castor oil, to produce an ester (product 1) (Figure 1). The fluorescent-labeled product 1 was purified in a preparative chromatographic column. The TLC (Figure 2) image, revealed with UV light, indicated that a fluorescent product was obtained without contamination of the unbound rhodamine B. Thus, the purification process was effective in removing molecules of rhodamine B that did not react with the ricinolein from the crude fluorescent product 1. The presence of free rhodamine B in the final product could lead to release of the fluorescence from the nanocapsule and thus unreliable results. The several spots observed for the purified fluorescent product 1 were expected since castor oil is a mixture of triglycerides and also because the rhodamine B molecule can react with one, two, or three of the hydroxyl groups presented in the ricinolein residue, which could result in products with different polarities.

The FTIR and ^1^H-NMR spectra (Figure 3 and Additional file 1: Figure S1B) showed that the main structure of the raw castor oil was maintained after the reaction. No band characteristic of carboxylic acid was observed on the FTIR spectrum of the purified product (Figure 3), and the signal with a chemical shift of 2.3, characteristic of the hydrogen atoms of an ester, was maintained (Additional file 1: Figure S1B). This suggests that no hydrolysis of the ester bound occurred. ^1^H-NMR spectrum of the fluorescent product 1 showed signals with chemicals shifts higher than 5.8 and an AB system corresponding to the hydrogen atoms of the aromatic ring of rhodamine B residue. However, as previously reported, the sensitivity of FTIR and ^1^H-NMR techniques can be not sufficient to detect some functional groups or the protons of the dye due to their small contribution compared to the contribution of the functions and hydrogen atoms of the oil residue [12,28]. Up to this point, the results (TLC, FTIR, and ^1^H-NMR) indicate that the functional carboxylic group of rhodamine B was bound to the ricinolein presented in the castor oil and that a fluorescent oily product was obtained presenting good purity regarding the presence of unbound rhodamine B.

UV-vis and fluorescence spectroscopy showed that the product 1 obtained presents maximum absorption (*λ*_max-ab_ = 519 nm) in the green region of the optical spectrum and maximum emission (Figure 4) in the yellow-orange region (567 nm). The results for the SEC analysis of the purified product 1 were consistent with the values obtained for the raw castor oil, demonstrating that the hydrodynamic volume and the size chain distribution were not modified after rhodamine B coupling to the product. The quantitative analysis of the amount of rhodamine B bound to the product indicated a concentration of bound dye of 0.517 ± 0.096 μmol per g of fluorescent oily product (*n* = 3). This corresponds to 1 rhodamine residue for 1,150 molecules of the product.

The rhodamine-labeled triglyceride was used to prepare fluorescent NC formulations with Eudragit RS100 or Eudragit S100, providing cationic and anionic particles, respectively. Fluorescent LNC were also prepared with the rhodamine-labeled product using poly(ϵ-caprolactone) as the polymer. The liquid portion of the nanocapsule core was composed of fluorescent triglyceride (10%) and CCT (90%) (Table 1). In the LNC-PCL formulations, the liquid portion was 160 μL/10 mL of suspension corresponding to approximately 1.52 mg of fluorescent product per mL of suspension, while in NC-RS100 and in NC-S100, the liquid portion was 333 μL/10 mL of suspension corresponding to approximately 3.15 mg of fluorescent product per mL of suspension. It is important to note that the amount of rhodamine-labeled triglyceride can be increased or decreased, according to the needs of the study. The pH of the nanocapsule formulations (Table 1) was slightly acid and similar to the values previously reported for formulations prepared without the fluorescent-labeled oil [26,29]. The size distribution profiles (Figure 5) and the *D*_4.3_, SPAN, *z*-average, PDI, and zeta potential values for the formulations containing the fluorescent product 1 (Table 1) did not differ considerably from those observed for non-fluorescent formulations [25-27].

The zeta potential values for the formulations prepared with the fluorescent product 1 (Table 1) showed values approximately closed to those previously reported for the similar formulations prepared without the dye-labeled oil [25-27]. The electrokinetic behavior of colloids is related to the movement of ionic solutions near charged interfaces [30]. The carboxylic acids, as pendant groups in Eudragit S100 or as terminal groups in PCL_116_, are in an acid-base balance at the particle-water interface producing carboxylate functions that react with NaCl forming the electrical double layer responsible for the eletrokinetic behavior of NC-S100 and LNC-PCL. On the other hand, the NC-RS has a polymer wall of poly(ethyl acrylate-co-methyl methacrylate-co-trimethylammonioethyl methacrylate chloride), whose monomer units are at 1:2:0.1 proportions. In this way, the trimethylammonioethyl moiety has a quaternary nitrogen giving to the particle-water interface a positive charge. The electrokinetic properties of NC-RS are related to the positive surface potential that those nanocapsules present after dilution in 10 mmol L^-1^ NaCl aqueous solution. Considering that all formulations contain polysorbate 80, the mechanism of stabilization of those colloids is not exclusively based on the electrical repulsion of the particles since the steric hindrance effect of the surfactant plays an important role [31-33]. Then, even though the zeta potential values are near zero for all formulations, the colloidal turbid solutions have an adequate kinetic stability for the purpose of drug delivery. The NC-RS100 and NC-S100 formulations presented higher concentrations of particles (approximately 1.7-fold and 1.4-fold, respectively) than LNC-PCL (*P* < 0.05). This result was expected since the volumetric fraction of the dispersed phase in these formulations is higher than that of LNC-PCL, and the *z*-average values obtained for each formulation were similar [8].

The fluorescence spectroscopy analysis of the fluorescent nanocapsules and fluorescent lipid-core nanocapsules showed that the fluorescence property is maintained after the preparation of these formulations (Figure 6). The difference in the fluorescence intensity on comparing the NC-RS100 and NC-S100 formulations with LNC-PCL was expected since the concentration of fluorescent product in these formulations varied (approximately 3.15 mg of product 1/mL of suspension for NC-RS100 and NC-S100 and approximately 1.52 mg of product 1/mL of suspension for LNC-PCL) (Figure 6). In the undiluted/unextracted samples of the formulations, it was seen that the bathochromic (7 nm) shift for the *λ*_max_**-**_em_ value in the emission spectrum of the NC-S100-1 formulation was accompanied by a hyperchromic shift (52 a.u.) when compared to the NC-RS100-1 formulation, which contains the same quantity of fluorescent product, probably due to protonation of the amino group of rhodamine B, as the pH of this formulation was the lowest among the formulations (3.50 ± 0.09). As previously reported, rhodamine B has an equilibrium of isoforms, lactonic and the zwitterionic isomers [34]. The zwitterion isomer can be protonated more than once due to the presence of two amino groups [34]. A hypochromic shift was observed in the emission spectra of the undiluted/unextracted samples of the LNC-PCL-1 (114 a.u.), NC-RS100-1 (230 a.u.), and NC-S100-1 (178 a.u.) formulations compared to the spectrum of the solutions containing the same quantity of the CCT/fluorescent oily product mixture in ACN [solution 1 (1.52 mg/mL) and solution 2 (3.15 mg/mL)] (Figure 6A,B). Unsurprisingly, in the case of the samples containing the CCT/fluorescent oily product mixture (Figure 6C,D), the results for the fluorescence intensity of the diluted/extracted samples of the formulations showed greater similarity when compared to the undiluted/unextracted samples. The previously observed hypochromic shift did not occur and a small hyperchromic shift occurred, especially for NC-RS100-2 (24 a.u.) and NC-S100-2 (27 a.u.). Therefore, these changes in the fluorescence intensity of the undiluted/unextracted samples are probably related to the volume fraction of particles in the dispersed phase of the formulation leading to phenomena such as the inner filter effect, where the presence of other compounds can partially absorb the emission energy, and they were not sufficiently reduced even with the use of a triangular cuvette [35,36].

To demonstrate the applicability of the synthesized fluorescent triglyceride (product 1) to the identification of particles containing this compound in image studies, a cell uptake study was performed. It was possible to observe red fluorescence in the cells treated with the fluorescent nanoparticles (Figure 7). The red fluorescence was very close to the cell nucleus suggesting that the particles are located inside the cells. Martins and co-workers [37] have reported the uptake of solid lipid nanoparticles (SLN) stabilized with polysorbate 80 by THP1-derived macrophages. The authors loaded the SLN with a green fluorescent dye and evaluated the particle uptake by fluorescence microscopy. In a recent study, our research group demonstrated the uptake of LNC-PCL, also stabilized with polysorbate 80, by macrophages isolated from BALB/c mice [11]. In this case, the LNC-PCL particles were prepared with the polymer chemically bound to rhodamine-B and non-labeled oil. The results reported herein reinforce these findings and can demonstrate the applicability of the use of the fluorescent triglyceride to localize particles in biological studies with the advantage of allowing the development of tracking systems with surfaces exhibiting a variety of chemical natures. In a forthcoming publication, the applicability of this product to tracking particle skin penetration and also particle uptake by skin cells, considering the influence of the particle surface properties, will be demonstrated.

Recently, in an *in vivo* study with rats implanted with glioma tumors, it was showed that, after 10 days of treatment, the group of animals treated with indomethacin loaded in LNC (IndOH-LNC) particles presented a higher concentration of the drug in the cerebral tissue and, more specifically, in the tumor hemisphere compared to the group which received the free drug [2]. The tumor size of the groups treated with IndOH-LNC [2] or *trans*-resveratrol loaded in LNC (*t*-resv-LNC) [38] particles was significantly reduced when compared to the groups treated with the free drug. A similar profile of higher drug concentration in the brain compared to the free drug was observed in a biodistribution study in rats treated with *trans*-resveratrol or *t*-resv-LNC particles [39]. Based on these findings, it is suggested that LNC particles are able to target the drug to the brain tissue and reduce the tumor size. The synthesis of fluorescent materials for the preparation of fluorescent dye-labeled nanocapsules, such as the fluorescent polymer [12] and the fluorescent triglyceride, product 1 (as reported herein), could also be useful for tracking the pathway of the LNC particles and/or their uptake in cells, for instance, in experiments similar to those cited here. Therefore, the labeled nanoparticles may be used to find the final destiny of the particles after *in vitro* and *in vivo* treatments.

## Conclusions

A fluorescent oily product, rhodamine-labeled triglyceride, was obtained without unbound rhodamine B. The product was used to prepare fluorescent polymeric nanocapsules with cationic or anionic surface charges. The results obtained for the physicochemical characterization of the fluorescent-labeled nanocapsules and fluorescent-labeled lipid-core nanocapsules were similar to those previously reported for formulations prepared without the fluorescent product indicating that the labeling did not affect the characteristics of the nanocarriers. Thus, our results indicate the possibility of obtaining versatile dye-labeled polymeric vesicular nanocarriers, with surfaces exhibiting different chemical natures, applying the strategy of fluorescent dye labeling of the triglyceride which forms the oily inner core of the nanocapsules. This fluorescent dye-labeled triglyceride could be used for particle localization in biological studies with the advantage among other fluorescent materials that any carrier that contains a triglyceride in its formulation composition can be obtained and tracked.

## Competing interests

The authors declare that they have no competing interests.

## Authors’ contributions

LAF carried out the synthesis and characterization of the fluorescent triglyceride and the preparation and characterization of the fluorescent nanoparticles, performed the cell uptake and the fluorescence microscopy studies, and performed the interpretation of data and manuscript writing. RVC participated in the synthesis and characterization of the fluorescent triglyceride and contributed to the design of experiments, interpretation of data, and manuscript drafting. JFB participated in the characterization of the fluorescent triglyceride and in the preparation and characterization of the fluorescent nanoparticles. FF carried out the cell culture and helped in the design and performance of the cell uptake and the fluorescence microscopy studies. AMOB conceived the study regarding the cell culture, cell uptake, and fluorescence microscopy. SSG conceived the study regarding the nanoparticle physico-chemical characterization and participated in the interpretation of data. ARP conceived the study and participated in its design, coordination, and result interpretations. All authors read and approved the final manuscript.

## Supplementary Material

Additional file 1 **Supplementary material.** Proton nuclear magnetic resonance of product 1. Click here for file
