# Supplementary material for: Labeling the oily core of nanocapsules and lipid-core nanocapsules with a triglyceride conjugated to a fluorescent dye as a strategy to particle tracking in biological studies
Source: Nanoscale Res Lett. 2014 May 13;9(1):233. doi: 10.1186/1556-276X-9-233 (PMC4045892; doi:10.1186/1556-276X-9-233)
Supplement: Additional file 1 — Supplementary material. Proton nuclear magnetic resonance of product 1. [file 1556-276X-9-233-S1.docx]

Supplementary material

Labeling the oily core of nanocapsules and lipid-core nanocapsules with a triglyceride conjugated to a fluorescent-dye as a strategy to particle tracking in biological studies

Luana Almeida Fiel,*^a,^** Renata Vidor Contri,*^a^* Juliane Freitas Bica,*^b^* Fabrício Figueiró,*^c^* Ana Maria Oliveira Battastini,*^c^* Sílvia Stanisçuaski Guterres*^a^* and Adriana Raffin Pohlmann*^a,b^*

________

^1^H-NMR spectra of the samples previously dissolved in deuterated chloroform were obtained using a Varian^®^ INOVA-300 spectrometer operating at 400 MHz at 20 ^o^C. The proton signal of residual CHCl_3_ (7.27 ppm) was used as the internal reference. Multiplicity abbreviations: multiplet, *m*.

^1^H-NMR (δ, ppm) CDCl_3_: 0.86 (*m*, CH_3_ at the end of the chain), 2.03 (*m*, CH_2_ at the middle of the chain), 2.28-2.31 (*m*, CH_2_ at positions 1, 2 and 4), 5.9-7 (CH of aromatic ring).


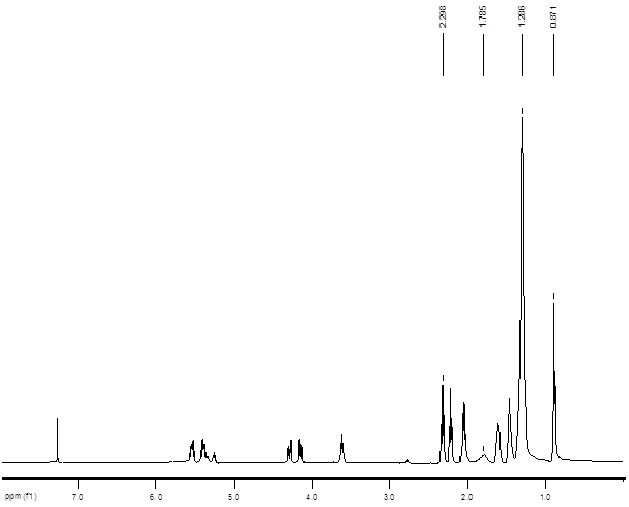

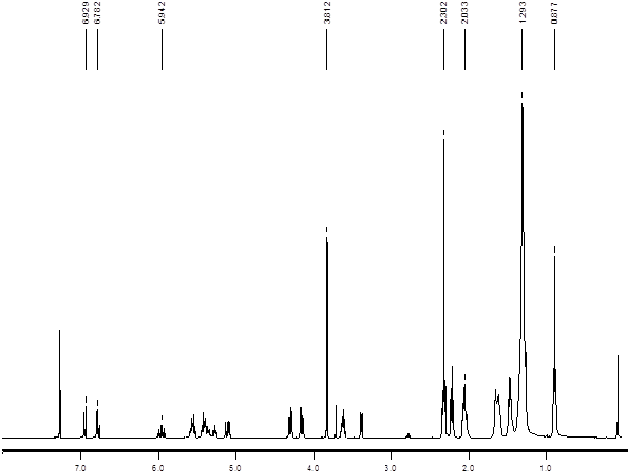


(B)

(A)

**Fig. S1** 1H-nuclear magnetic resonance spectra of (A) raw castor oil. and (B) puriﬁed fluorescent product **1.**
